# Using HSV-TK/GCV suicide gene therapy to inhibit lens epithelial cell proliferation for treatment of posterior capsular opacification

**Published:** 2011-01-27

**Authors:** Yong-Xiang Jiang, Yi Lu, Tian-Jing Liu, Jin Yang, Yan Chen, Yan-Wen Fang

**Affiliations:** 1 Department of Ophthalmology, Eye and ENT Hospital, Fudan University, Shanghai, China; 2 Genetic Engineering Group, Institute of Biochemistry and Cell Biology, Chinese Academy of Science, Shanghai, China

## Abstract

**Purpose:**

To establish a novel, targeted lentivirus-based HSV-tk (herpes simplex virus thymidine kinase)/GCV (ganciclovir) gene therapy system to inhibit lens epithelial cell proliferation for treatment of posterior capsular opacification (PCO) after cataract surgery.

**Methods:**

An enhanced Cre recombinase (Cre/loxP) system with a lentiviral vector expressing Cre under the control of the lens-specific promoter LEP503 (Lenti-LEP503-HSVtk-Cre [LTKCRE]) was constructed, as well as another lentiviral vector containing a switching unit. The latter vector contains a stuffer sequence encoding EGFP (Lenti-hPGK-Loxp-EGFP-pA-Loxp-HSVtk [PGFPTK]) with a functional polyadenylation signal between two loxP sites, followed by the herpes simplex virus thymidine kinase (*HSV-tk*) gene, both under the control of the human posphoglycerate kinase (*hPGK*) promoter. Expression of the downstream gene (*HSV-tk*) is activated by co-expression of Cre. Human lens epithelial cells (HLECs) or retinal pigmental epithelial cells (RPECs) were co-infected with LTKCRE and PGFPTK. The inhibitory effects on HLECs and RPECs infected by the enhanced specific lentiviral vector combination at the concentration of 20 µg/ml GCV were assayed and compared.

**Results:**

The specific gene expression of Cre and *HSV-tk* in HLECs is activated by the LEP503 promoter. LTKCRE and PGFPTK co-infected HLECs, but not RPECs, expressed high levels of the HSV-tk protein. After 96 h of GCV treatment, the percentage of apoptotic HLECs infected by the enhanced specific lentiviral vector combination was 87.23%, whereas that of apoptotic RPECs was only 10.12%. Electron microscopy showed that GCV induced apoptosis and necrosis of the infected HLECs.

**Conclusions:**

The enhanced specific lentiviral vector combination selectively and effectively expressed *HSV-tk* in HLECs. A concentration of 20 µg/ml, GCV is effective against the proliferation of HLECs in vitro. This cell-type-specific gene therapy using a Cre/loxP lentivirus system may be a feasible treatment strategy to prevent PCO.

## Introduction

Posterior capsular opacification (PCO) caused by proliferation of residual epithelial cells over the lens equator and onto the posterior lens capsule [1] is the leading cause of visual impairment and blindness after cataract surgery [2-4]. There are currently no effective means by which to eradicate the residual lens epithelial cells during the operation [5,6]. In spite of improvements in the basic research on development of cataracts, surgical techniques, and the material or the design of the intraocular lens, the incidence of PCO is still 8~34.3% in adults, and nearly 100% in children [7-10]. One new promising approach for treatment of PCO is a gene therapy system uses a so-called suicide gene, the herpes simplex virus type 1 thymidine kinase (*HSV-tk*) gene, and the anti-herpes nucleoside analog drug, ganciclovir (GCV) [11-13]. In this system, GCV is phosphorylated by HSV-tk into a deoxynucleotide analog that becomes incorporated into DNA during strand replacement in proliferating cells, where it acts as a chain terminator of DNA synthesis and kills the dividing cells [14-16].

However, the standard HSV-tk/GCV system is driven by a constitutive promoter [11,12], and was shown to not only cause the death of the lens epithelial cells, but also of the corneal endothelial cells and the iris pigmental epithelial cells [11]. This cytotoxic side effect is the main obstacle for further clinical application of this system. However, the use of a lens-specific promoter would greatly benefit the application of this system for treatment of PCO. *LEP503* (lens epithelium gene product 503), which is a highly conserved gene involved in lens epithelial cell differentiation in different vertebrate species, is localized in the epithelial cells along the entire anterior surface of the lens. *LEP503* may be an important lens epithelial cell gene involved in the processes of epithelial cell differentiation [17]. The expression of *LEP503* is highly restricted to lens epithelial cells in vivo, and 2.5-kb flanking sequence-directed high-level promoter activity in lens epithelial cells,but not in other cell types [18]. Malecaze et al. [19] found that *LEP503*, *MIP* (major intrinsic protein), and Filensin promoters induced strong lens-specific expression of a reporter gene in human lens cells. The efficacy of *LEP503* promoters for a reporter gene expression is restricted to the residual lens cells post-PCO. We have found that human cytomegalovirus (CMV) promoter driven *HSV-TK* can inhibited the HLEC proliferation, though this system has no cell specification [20,21]. To avoid the toxic effects of the constitutive promoter on the surrounding normal cells, we constructed the HSV-tk/GCV vector with the lens-specific promoter *LEP503* (Lenti-LEP503-EGFP-HSVtk [LGFPTK]) and found that it can specifically express the HSV-tk protein in lens epithelial cells. However, the promoter inserted in this vector cannot provide high levels of expression. Indeed, the transduction efficiency of this vector was only 17.32%. Because the expression of *HSV-tk* induced by the lens-specific promoter *LEP503* was lower than that of the CMV promoter, we reasoned that it would not effectively inhibit the proliferation of lens epithelial cells.

It was recently reported that gene therapy using the Cre/loxP system greatly enhances the expression of the *HSV-tk* gene [15,22-24], especially that transduced by adenoviruses under the control of tissue-specific promoters such as the carcinoembryonic antigen (*CEA*) promoter and thyroglobulin promoter [24,25]. In these studies, the sensitivity of tumor cells to GCV was increased up to 5 to 10-fold compared with sensitivity in the presence of the promoter alone [26].

In the present study, to enhance the expression of the lens-specific promoter *LEP503*, we employed the HSV-tk/Cre/loxP system for gene therapy, targeting human lens epithelial cells (HLECs). Cre-loxP system-mediated lentiviruses, bearing an ON/OFF switching unit for activation by Cre recombinase, were used. We constructed an enhanced specific lentiviral vector combining two vectors: one is a regulatory vector (Lenti-LEP503-HSVtk-Cre [LTKCRE]) that expresses the Cre recombinase gene under the control of the *LEP503* promoter, while the other is a target vector(Lenti-hPGK-Loxp-EGFP-pA-Loxp-HSVtk [PGFPTK]). The switching unit in the lentiviral vector contains a stuffer sequence encoding enhanced green fluorescent protein (EGFP) with a functional polyA sequence between the strong human posphoglycerate kinase (*hPGK*) promoter and the inserted *HSV-tk* fragment, thereby inducing *EGFP* gene expression without *HSV-tk* expression. A pair of loxP sites flanking the stuffer sequence allows its excision by the Cre recombinase, leading to expression of the *HSV-tk* sequence instead of *EGFP*.

**Figure 1:**
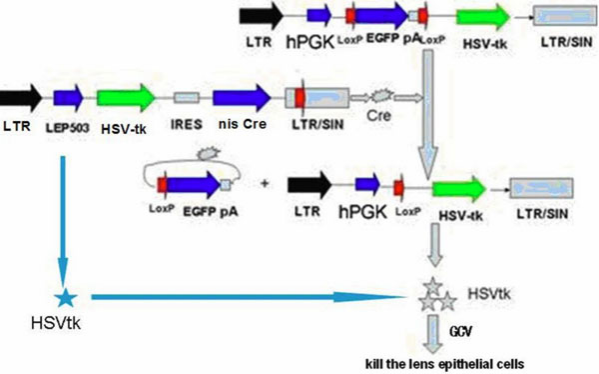
The mechanism of the Cre/loxP system combined with the lens-specific promoter *LEP503* based on lentiviral vectors.

Thus, we used the regulatory vector (LTKCRE) to express the HSV-tk protein and Cre recombinase after infection of HLECs by the two lentiviral vectors. The induced Cre recombinase should excise the functional polyA sequence interposed between the two loxP sites in the target vector (PGFPTK). Consequently, *HSV-tk* gene expression would be driven by the stronger *hPGK* promoter after activation by Cre recombinase. The amount of HSV-tk expressed by the Cre/loxP system-mediated lentiviruses should be greater than that expressed by the lentiviruses driven only by lens-specific promoter *LEP503* (Figure 1). We then evaluated the efficacy of gene therapy against proliferation of HLECs using these vectors with the Cre/loxP system and GCV treatment. Our findings provide experimental evidence for further development of this potential therapy for clinical treatment of PCO.

## Methods

### Construction of the enhanced specific lentiviral vector combination

The enhanced specific lentiviral vector combination includes two lentiviral vectors. One is a regulatory vector (LTKCRE), while the other is a target vector (PGFPTK). Cre recombinase was cloned from the Lenti-Cre plasmid by PCR, and sub-cloned into a T vector, which was double-digested by EcoRI and SalI enzymes. At the same time, the LGFPTK plasmid (constructed in our laboratory, unpublished data) was also digested by EcoRI and SalI enzymes. The two digested sections were ligated together to construct the regulatory vector LTKCRE. Loxp-polyA-Loxp was artificially synthesized with a PmeI enzyme site at the 5′ end of the polyA, and was inserted into a T vector along with *EGFP*, which had PmeI enzyme-digested sites at both terminal ends, resulting in the Loxp-EGFP-pA-Loxp vector. The Loxp-EGFP-pA-Loxp vector and plasmid PRRL were both double-digested by BamHI and SalI enzymes and then directionally combined. IRES-HSVtk was cloned by PCR with SalI restriction sites at both ends and inserted into the SalI-digested construction vector to produce the target vector PGFPTK.

### Production of lentiviral vectors

Replication-defective lentiviral particles pseudotyped with a VSV-G envelope were produced by three plasmid transient transfections of 293T cells, as previously described [20], with 20 μg of one of the gene transfer constructs (LTKCRE, PGFPTK), 12 μg of psPAX2, and 5 μg of pMD2.G using a calcium phosphate transfection kit (Gibco-BRL, Gaithersburg, MD). The transfection medium was replaced with fresh culture medium after 14 to 16 h. The conditioned medium was collected after another 24 h, cleared by low-speed centrifugation, and filtered through 0.45-mm filters (Nalgene, Rochester, NY). The virus was collected by ultracentrifugation for 90 min at 80,000× g at 4 °C. The pellet was resuspended in 1 ml phosphate buffered saline (PBS).

### FACS and PCR analysis of the infection efficiency of the lentiviral infection

To determine the infection efficiency of the enhanced specific lentiviral vector combination (PGFPTK and LTKCRE), EGFP expression was visualized by fluorescence microscopy and analyzed by FACS. Because the regulatory vector LTKCRE does not express EGFP, we detected the infection efficiency of LGFPTK. The HLEC line (SRA 01/04, cell line transformed by large T antigen) and retinal pigmental epithelial cells (RPECs, A PRE-19) were obtained from the ATCC (Manassas, VA ) and cultured in DMEM with 10% FBS. The HLECs and RPECs were plated separately at 2×10^5^ cells/well in 6-well plates for 24 h, and then LGFPTK and PGFPTK at a multiplicity of infection (MOI) of 20 were added. Polybrene (8 μg/ml) was added to the two sets of cultures, and the infected HLECs and RPECs were cultured for 72 h. EGFP expression in the HLEC group was visualized by fluorescence microscopy and analyzed by FACS. The primer sequences were as follows: *EGFP* (upstream 5′-cga gct gga cgg cga cgt aaa c-3′; downstream 5′-gcg ctt ctc gtt ggg gtc ttt g-3′) and glyceraldehyde-3-phosphate dehydrogenase (*GAPDH*; upstream 5′-aac gag cgg ttc cga tgc cct gag-3′; downstream 5′-tgt cgc ctt cac cgt tcc agt t-3′). Cycling conditions for amplification were: 94 °C for 5 min; 28 cycles at 94 °C for 1 min, 58 °C for 1 min, and 72 °C for 1 min; and finally, 72 °C for 10 min. The expected length of the EGFP PCR products was 597 bp. Differences in expression were normalized to the *GAPDH* signal (590 bp). Ten microliters from each RT–PCR product was electropheresed on a 1.5% agarose gel containing 0.5 µg/ml of ethidium bromide.

### The expression of EGFP in HLECs and RPECs infected by the enhanced specific lentiviral vector combination

To analyze the expression of *EGFP*, the HLECs and RPECs were plated separately at 2×10^5^ cells/well in 6-well plates, and LTKCRE (MOI=100), PGFPTK (MOI=20), or the enhanced specific lentiviral vector combination (PGFPTK [MOI=20] and LTKCRE [MOI=100]) were added. Polybrene (8 μg/ml) was added to all cultures, and the infected HLECs and RPECs were cultured for 72 h. EGFP expression in the HLEC group and RPEC group were visualized by fluorescence microscopy and analyzed by FACS.

### Western blotting analysis

To analyze the protein expression of Cre, EGFP, and HSV-tk from the lentiviral vectors, 1×10^6^ HLECs were seeded in 25 mm^2^ cell culture flasks for 24 h and then LTKCRE (MOI=100), PGFPTK (MOI=20), or the enhanced specific lentiviral vector combination (PGFPTK [MOI=20] and LTKCRE [MOI=100]), was added. At 72 h after the infection, the cells were boiled with 6× loading buffer for 10 min, and the cell proteins (20 μg) were separated in SDS–PAGE gels, followed by blotting onto polyvinylidene difluoride (PVDF) membranes. The anti-β-actin antibody from Santa Cruz Biotechnology (1:5000, Heidelberg, Germany) and anti-Cre, anti-EGFP, and anti-HSV-tk antibodies from Sigma-Aldrich (1:2,000; St. Louis, MO) were used to detect the lentivirally expressed Cre, EGFP, and HSV-tk proteins. The membranes were then incubated with a horseradish peroxidase-conjugated goat anti-mouse or anti-rabbit secondary antibody (Amersham, Arlington Heights, IL) and visualized using a chemiluminescence system (Amersham™ ECL).

### Cytotoxicity of GCV

To evaluate the cytotoxicity of GCV (InvivoGen, SanDiego, CA) treatment, apoptotic levels of HLECs and RPECs were evaluated. HLECs and RPECs were plated separately in 96-well plates at a density of 5×10^4^ cells/well and incubated for 24 h. Cells infected with Lenti-hPGK-EGFP-HSVtk (MOI=20), LTKCRE (MOI=100), or the enhanced specific lentiviral vector combination (PGFPTK [MOI=20] and LTKCRE [MOI=100]) served as the therapeutic groups, and cells infected with Lenti-IRES-EGFP (MOI=20) served as the control group. After 24 h, the four groups of cells were treated with GCV at increasing concentrations (0, 10, 20, 30, 40, and 50 μg/ml) and incubated at 37 °C in 5% CO_2_ for 4 days. Each group was assayed in triplicate for each concentration. Apoptosis of infected cells after the 4-day incubation was evaluated quantitatively by measuring phosphatidylserine externalization, an early apoptosis-related event. This was performed by fluorescence staining using the annexin V(blue)-PI staining kit (Roche, Basel, Switzerland) as per the manufacturer’s recommendations.

To further evaluate the cytotoxicity of GCV treatment, HLECs were plated in 96-well plates at a density of 5×10^4^ cells/well and incubated for 24 h. Cells infected with Lenti-hPGK-EGFP-HSVtk (MOI=20), LTKCRE (MOI=100), or the enhanced specific lentiviral vector combination [(PGFPTK (MOI=20) and LTKCRE (MOI=100)] served as therapeutic groups, and cells infected with Lenti-IRES-EGFP (MOI=20) served as a control group. After 24 h, the four groups were treated with GCV at increasing concentrations (0, 10, 20, 30, 40, and 50 μg/ml) and incubated at 37 °C in 5% CO_2_ for 1 to 4 days. Cell viability of the four groups was determined using the Cell Counting kit-8 Cell Proliferation Assay (Dojindo, Kumamoto, Japan) after 24 or 96 h. At each time point, cells were assayed in triplicate for each concentration. The cytotoxic effect was indicated as the percentage of surviving cells (ratio of surviving cells after treatment and without treatment) using the following formula: cell viability=(absorption of lenti-HSV-tk-EGFP−absorption of background)/(absorption of lenti-EGFP absorption of background) × 100%. A p value of <0.05 was considered statistically significant.

### Electron microscopy

HLECs (1×10^6^/well, 6-well plates) infected with the enhanced specific lentiviral vector combination (PGFPTK [MOI=20] and LTKCRE [MOI=100]) served as a therapeutic group; HLECs infected with Lenti-IRES-EGFP (MOI=20) served as a control group. After treatment with GCV (20 μg/ml) for 96 h, cells were collected with a cell scraper, followed by three 10-min washes in PBS at room temperature. The cell pellet was fixed with 5% glutaraldehyde for at least 30 min, fixed in PBS containing 1% osmium tetroxide for 1 h, dehydrated in grading ethanol, and embedded in resin. Sections (100 nm thick) were counterstained with uranyl acetate and lead citrate and examined by transmission electron microscopy (H-7650; Hitachi, Tokyo, Japan). The characteristic morphologic changes of infected HLECs were recorded, including chromatin condensation, plasma membrane blebbing, cell shrinkage, and fragmentation into membrane bound bodies.

### Statistical analysis

Treatments were examined and analyzed statistically using the SPSS statistical package, version 11.0 (SPSS Inc., Chicago, IL) for Windows. Data are expressed as mean±standard deviation of separate experiments. ANOVA and *t* tests were performed to assess the statistical significance between different groups. A p value of <0.05 was considered statistically significant.

## Results

### The *LEP503* promoter drives *EGFP* expression specifically but relative weakly in HLECs

**Figure 2:**
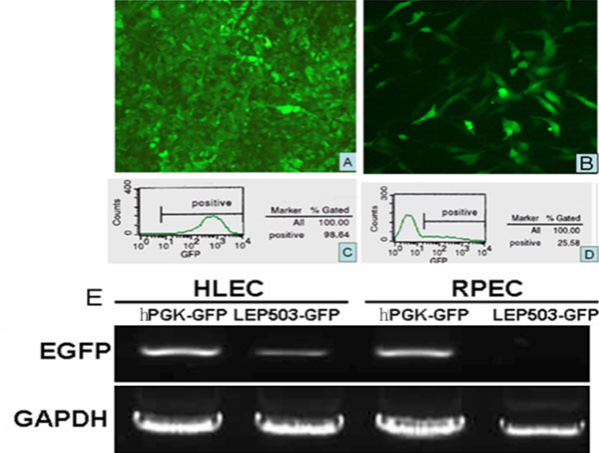
*LEP503* promoter specifically, but relative weakly, induced expression of *EGFP* in HLECs. The hPGK promoter and *LEP503* promoter-induced *EGFP* expression in HLECs was detected by fluorescence microscopy (**A**, **B**) and FACS (**C**, **D**). RT–PCR analysis of *EGFP* expression showed that PGFPTK can be expressed well in HLECs and RPECs. However, LGFPTK was specifically expressed in HLECs and not in RPECs (**E**).

In comparing HLECs at 3 days post-infection by two lentiviral vectors (MOI=20), a stronger fluorescent signal was detected after PGFPTK infection (Figure 2A). However, a low level of EGFP expression was detected in the HLECs infected by LGFPTK under a fluorescence microscope (Figure 2B). FACS detections of positive EGFP were 98.64% (Figure 2C) and 25.58% (Figure 2D) by PGFPTK and LGFPTK infection, respectively. This demonstrated that the constitutive promoter *hPGK* was stronger than the *LEP503* promoter in HLECs. To detect the specificity of the *LEP503* promoter in HLECs, we analyzed the expression of EGFP in HLECs and RPECs, which are both important cells related to visual function in eye. RT–PCR results showed that PGFPTK expressed well in both HLECs and RPECs. However, LGFPTK was only expressed in HLECs, as there was no detectable *EGFP* in RPECs (Figure 2E). This result demonstrated that the *LEP503* promoter can specifically induce HSVtk-EGFP expression in HLECs with lower efficiency than the constitutive promoter *hPGK*.

### The enhanced specific lentiviral vector combination drives high HSVtk protein expression in HLECs

**Figure 3:**
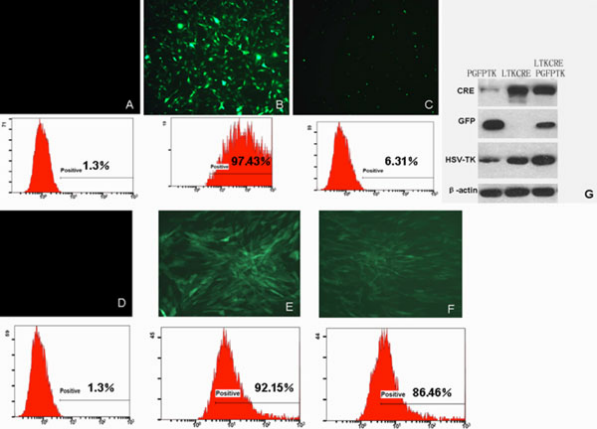
The enhanced specific lentiviral vector combination induces high HSVtk protein expression in HLECs. After transfection with LTKCRE, PGFPTK, and the enhanced specific lentiviral vector combination, the HLECs (**A**-**C**) and RPECs (**D**-**F**) were observed under a fluorescence microscope and FACS at 72 h. Cells infected with LTKCRE did not express EGFP (**A**, **D**). The transduction efficiency of PGFPTK measured by FACS was 97.43% and 92.15% in HLECs and RPECs, respectively (**B**, **E**). Meanwhile the transduction efficiency of the enhanced specific lentiviral vector combination in HLECs and RPECs was 6.31% and 86.46%, respectively. Cre, EGFP, and HSV-tk protein expressions in HLECs were analyzed by western blot for cells infected by different lentiviral vectors (**G**).

The enhanced specific lentiviral vector combination was composed of LTKCRE and PGFPTK. The mechanism of this expression system is shown in Figure 1. LTKCRE specifically expressed the HSV-tk protein and Cre recombinase in HLECs. PGFPTK could express *EGFP* well, but not *HSV-tk* with the polyA behind *EGFP*. When both of them were expressed in HLECs, *LEP503* specifically induced the Cre recombinase (LTKCRE) to excise the functional polyA sequence interposed between the two loxP sites in the target vector (PGFPTK). Then, *HSV-tk* gene expression could be driven by the stronger *hPGK* promoter after activation by the Cre recombinase. The HSV-tk expressed by this vector combination was greater than that expressed in the presence of the promoter alone (LTKCRE). To estimate the transduction efficiency and specificity of the lentiviral vectors, we infected HLECs (Figure 3A-C) and RPECs (Figure 3D-F). The regulatory vector LTKCRE did not express EGFP (Figure 3A,D). The transduction efficiencies of PGFPTK in HLECs and RPECs measured by FACS analysis were 97.43% and 92.15%, respectively (Figure 3B,E). Meanwhile, the expression of EGFP was drastically reduced to 6.3% by this enhanced specific lentiviral vector combination in HLECs (Figure 3C), and there was no significant change in expression between the lentiviral vector combination applied to RPECs (86.46%; Figure 3F) and that from the infection of PGFPTK in RPECs (Figure 3E). We analyzed the expressions of Cre, EGFP, and HSV-tk by western blot analysis (Figure 3G). As expected, Cre was expressed only in the HLECs infected by LTKCRE and the enhanced specific lentiviral vector combination. No EGFP expression was observed in the HLECs infected by LTKCRE, whereas EGFP expression in HLECs infected by PGFPTK was stronger than infection by the enhanced specific lentiviral vector combination. For HSV-tk, HLECs infected by the enhanced specific lentiviral vector combination showed stronger expression than by LTKCRE, whereas there was very weak expression in the HLECs infected by PGFPTK.

### Apoptosis of HLECs treated with GCV in different lentiviral vector groups

**Figure 4:**
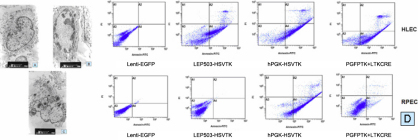
Apoptosis profiles of HLECs at 96 h after infection by different vectors and treated with GCV at 20 µg/ml. Electron microscopy showing subcellular structures of infected HLECs. The Lenti-IRES-EGFP infected cell nuclei were intact (**A**; magnification, 4880×). The enhanced specific lentiviral vector combination infected HLECs appeared as apoptotic bodies (**B**; magnification, 6550×). Necrosis (cell swelling, rounded mitochondrion, some subcellular structures) was seen in the cytoplasm (**C**; magnification, 4880×). The percentages of apoptotic HLECs and RPECs infected by vectors: Lenti-hPGK-EGFP-HSVtk=90.12% and 89.68%, respectively; LTKCRE=53.2% and 8.62%, respectively, the enhanced specific lentiviral vector combination=87.23% and 10.12%, respectively; Lenti-IRES-EGFP=0.96% and 0.75%, respectively.

The cell morphologic changes of the therapeutic group (the enhanced specific lentiviral vector combination) and the control group (Lenti-IRES-EGFP) were studied using transmission electron microscopy. Compared with the normal shapes and sizes of the cells in the control group (Figure 4A), the typical morphologic changes of apoptosis were seen in the enhanced specific lentiviral vector combination infected cells treated with 20 μg/ml GCV for 96 h (Figure 4B). The characteristics of apoptosis included cell shrinkage, chromatin condensation, nuclear fragmentation, and cytoplasm degradation. The nuclei were irregular and bulbous; at the same time, necrosis was seen in a few cells of the therapeutic group (Figure 4C). The cells were swollen with a collapsed membrane, loss of structural integrity, and round mitochondria. Intracellular contents were released, which indicated a typical pattern of cell necrosis. The electron microscopy results showed that GCV at a concentration of 20 μg/ml can induce apoptosis and necrosis of cells infected with the enhanced specific lentiviral vector combination.

Apoptotic levels of HLECs and RPECs were analyzed by FACS 96 h after infection in different lentiviral vector groups treated with GCV at the concentration of 20 µg/ml (Figure 4D). The percentage of apoptosis in the HLECs infected with the enhanced specific lentiviral vector combination (PGFPTK and LTKCRE) was 87.23%, and the percentage of apoptosis in the positive control vector (Lenti-hPGK-EGFP-HSVtk) group was 90.12%. The apoptotic level was somewhat lower, at 53.2%, in the cells infected individually with the vectors carrying the lens epithelial cell specific promoter (LTKCRE), while that of the negative control vector Lenti-IRES-EGFP was 0.96%. For the RPECs, the percentages of apoptosis in cells infected with different vectors were 10.12% (PGFPTK and LTKCRE), 89.68% (Lenti-hPGK-EGFP-HSVtk), 8.62% (LTKCRE), and 0.75% (Lenti-IRES-EGFP).

The levels of apoptosis in HLECs infected with the enhanced specific lentiviral vector combination were obviously higher than those in the LTKCRE-infected groups, and a specificity for HLECs over RPECs was also shown.

### Cytotoxicity of the HSV-tk/GCVsystem

To determine the effect of GCV on HLECs, the drug was added at increasing concentrations (from 0 to 50 μg/ml) to four groups of cells [infected with Lenti-hPGK-EGFP-HSVtk, LTKCRE, the enhanced specific lentiviral vector combination (PGFPTK and LTKCRE), and Lenti-IRES-EGFP], which were then incubated for 24 or 96 h. At each time point, the cytotoxicities of the cells were assayed in triplicate for each concentration using the Cell Counting Kit-8 (CCK-8).

**Figure 5:**
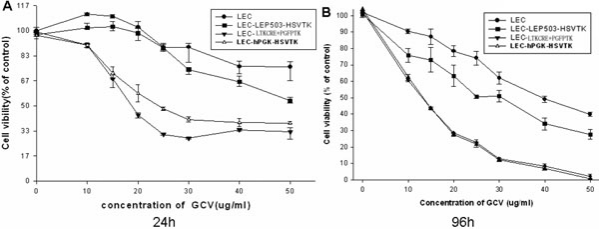
The dose and time dependency of the HSV-tk/GCV system to affect cell viability detected using CCK-8. Different concentrations of GCV were added to the HLECs infected by Lenti-IRES-EGFP, LTKCRE, the enhanced specific lentiviral vector combination, or Lenti-hPGK-EGFP-HSVtk for 24 h (**A**) or 96 h (**B**). The cell viability of the group treated with the enhanced specific lentiviral vector combination was significantly suppressed compared with that treated with LTKCRE.

The cell cytotoxicity gradually became more obvious with increasing GCV concentrations (Figure 5). The results indicated a dose-dependent effect on survival of the infected HLECs treated with GCV. At a concentration of 20 µg/ml after 96 h, there was no significant difference (p>0.05) in the cell viability between the enhanced specific lentiviral vector combination and the Lenti-hPGK-EGFP-HSVtk group. However, the cell viability with the enhanced specific lentiviral combination infection was significantly lower (p<0.05) than that of LTKCRE-infected cells. The cell viability of the enhanced specific lentiviral vector combination at 24 h was significantly higher (p<0.05) than that at 96 h.

## Discussion

Clinically, PCO is treated by Nd:YAG capsulotomy or secondary capsulotomy, which carries a risk of sight-threatening complications such as cystoid macular edema, retinal detachment, and increased intraocular pressure [3,4]. With the development of molecular biology, gene therapy for PCO in vitro has made further advances. Several studies have experimentally demonstrated the cytotoxic effects of the HSV-tk/GCV system on lens epithelial cells and showed that it effectively inhibits the proliferation of lens epithelial cells in vitro [11,13,19,25,26]. However, further application of HSV-tk gene therapy for PCO treatment in vivo is dependent on eliminating the toxic side effects on the cells surrounding the lens epithelial cells. The use of the lens-specific promoter *LEP503* may be a desirable strategy for targeted gene therapy for PCO. To increase the low expression activity of this tissue-specific promoter [27], we used the Cre/loxP system involving double lentivirus vectors in the present in vitro study.

In comparing HLECs infected by various vectors, no EGFP-positive cells could be seen in the regulatory vector LTKCRE-infected group, and very few were infected by the enhanced specific lentiviral vector combination expressed-EGFP; however, almost all of the HLECs in the targeted vector PGFPTK group expressed EGFP, as observed by fluorescent microscopy. Western blot analysis confirmed these EGFP expressions. For *HSV-tk*, there was weak expression in the HLECs infected by the targeted vector PGFPTK, while those infected by the regulatory vector LTKCRE showed stronger expression of *HSV-tk*. Finally, the strongest expression of *HSV-tk* was observed in HLECs infected by the enhanced specific lentiviral vector combination. Cre was only expressed in the HLECs infected by the regulatory vector LTKCRE and the enhanced specific lentiviral vector combination. These data demonstrated that the use of the constructed double lentiviral vector combination was successful in terms of the high level of the promoter expression. However, there was weak expression of the HSV-tk protein in HLECs infected by the targeted vector PGFPTK, suggesting that the efficacy of the functional polyA sequence needs further improvement.

This expression of HSV-tk in HLECs resulted in marked specific enhancement of cytotoxicity after exposure to GCV. Similar dose-dependent cytotoxic effects of GCV on infected HLECs, as well as in other virus-mediated systems, have been previously observed [11,20]. In our previous experiments, we found that 20 to 25 μg/ml of GCV was the best range of concentrations of GCV for inhibiting HSV-tk–positive cells without cytotoxicity to normal cells [20]. We evaluated the inhibitory effect of the HSV-tk/GCV system on HLECs through analysis of apoptosis and viability using CCK-8. After treatment of the cells with GCV at a concentration of 20 µg/ml for 96 h, the apoptosis rate detected by flow cytometry in the HLECs transduced by the enhanced specific lentiviral vector combination was high, at 87.23%. Additionally, the cell viability of the enhanced specific lentiviral vector combination at 96 h was significantly lower than that at 24 h, which indicated that apoptotic cells increased with prolonged incubation time due to an in vitro bystander effect of HSV-tk–expressing cells [20].

The double lentiviral vectors combination of the HSV-tk/GCV system effectively inhibited the proliferation of HLECs in our study. To verify our observations, electron microscopy and EGFP detection were also applied, which showed that the cytotoxic mechanism of the HSV-tk/GCV system includes apoptosis and necrosis of infected cells. The fact that the apoptosis rate was relatively high at 87.23% but not 100% is perhaps an advantage. That is, killing all residual lens epithelial cells may ultimately result in capsular defects since growth and/or maintenance of the capsule requires living lens cells, especially in children [28,29].

In addition to the high HSV-tk expression in the lens epithelial cells, the specific HSV-tk expression is another important index in the evaluation of the enhanced specific lentiviral vector combination. After the RPECs were infected with LGFPTK, RT–PCR analysis showed there was no detectable *EGFP* in the cells, while the infection of the targeted vector PGFPTK or the enhanced specific lentiviral vector combination resulted in nearly the same high level of *EGFP* positive cells in the two groups. These data indicated that there was no *HSV-tk* and Cre expression in RPECs under the control of the lens-specific promoter *LEP503*. After treatment of the cells with GCV (20 µg/ml), even for 96 h, there was little apoptotic effect on RPECs infected by the enhanced specific lentiviral vector combination. Therefore, the double lentiviral vectors combination as constructed would have restricted expression of *HSV-tk* in HLECs, thereby avoiding toxic effects to the surrounding normal cells.

In conclusion, the lentiviral vector combination using the Cre/loxP system can improve the expression of the *HSV-tk* gene driven by the lens-specific promoter *LEP503*. At a concentration of 20 µg/ml, GCV was effective against the proliferation of HLECs in vitro, but had little killing effect on RPECs. In future experiments, we will verify the inhibitory effects of this lentiviral vector combination on the lens capsule or PCO models.

## References

[1] Nishi O Posterior capsule opacification. Part 1: Experimental investigations. J Cataract Refract Surg 1999 25 106 17 9888086 10.1016/s0886-3350(99)80020-0

[2] Ashwin PT Shah S Wolffsohn JS Advances in cataract surgery. Clin Exp Optom 2009 92 333 42 19570151 10.1111/j.1444-0938.2009.00393.x

[3] Ozkurt YB Sengor T Evciman T Haboglu M Refraction, intraocular pressure and anterior chamber depth changes after Nd:YAG laser treatment for posterior capsular opacification in pseudophakic eyes. Clin Exp Optom 2009 92 412 5 19549226 10.1111/j.1444-0938.2009.00401.x

[4] Roh JH Sohn HJ Lee DY Shyn KH Nam DH Comparison of posterior capsular opacification between a combined procedure and a sequential procedure of pars plana vitrectomy and cataract surgery. Ophthalmologica 2010 224 42 6 19684427 10.1159/000234907

[5] Awasthi N Guo S Wagner BJ Posterior capsular opacification: a problem reduced but not yet eradicated. Arch Ophthalmol 2009 127 555 62 19365040 10.1001/archophthalmol.2009.3

[6] Yadav UC Ighani-Hosseinabad F van Kuijk FJ Srivastava SK Ramana KV Prevention of posterior capsular opacification through aldose reductase inhibition. Invest Ophthalmol Vis Sci 2009 50 752 9 19011011 10.1167/iovs.08-2322 PMC2832582

[7] Gu F Zhai H Li D Zhao L Li C Huang S Ma X A novel mutation in major intrinsic protein of the lens gene (MIP) underlies autosomal dominant cataract in a Chinese family. Mol Vis 2007 13 1651 6 17893667

[8] Rönbeck M Zetterstrom C Wejde G Kugelberg M Comparison of posterior capsule opacification development with 3 intraocular lens types: five-year prospective study. J Cataract Refract Surg 2009 35 1935 40 19878826 10.1016/j.jcrs.2009.05.048

[9] Vasavada AR Dholakia SA Raj SM Singh R Effect of cortical cleaving hydrodissection on posterior capsule opacification in age-related nuclear cataract. J Cataract Refract Surg 2006 32 1196 200 16857509 10.1016/j.jcrs.2006.03.017

[10] Vasavada AR Nihalani BR Pediatric cataract surgery. Curr Opin Ophthalmol 2006 17 54 61 16436925 10.1097/01.icu.0000193069.32369.e1

[11] Couderc BC de Neuville S Douin-Echinard V Serres B Manenti S Darbon JM Malecaze F Retrovirus-mediated transfer of a suicide gene into lens epithelial cells in vitro and in an experimental model of posterior capsule opacification. Curr Eye Res 1999 19 472 82 10550788 10.1076/ceyr.19.6.472.5284

[12] Malecaze F Couderc B de Neuville S Serres B Mallet J Douin-Echinard V Manenti S Revah F Darbon JM Adenovirus-mediated suicide gene transduction: feasibility in lens epithelium and in prevention of posterior capsule opacification in rabbits. Hum Gene Ther 1999 10 2365 72 10515456 10.1089/10430349950017013

[13] Wang B Weng J Effects of adenovirus-mediated HSV-tk/GCV system on lens epithelium. Zhonghua Yan Ke Za Zhi 2002 38 618 22 12487913

[14] Denny WA Prodrugs for Gene-Directed Enzyme-Prodrug Therapy (Suicide Gene Therapy). J Biomed Biotechnol 2003 2003 48 70 12686722 10.1155/S1110724303209098 PMC179761

[15] Maeda M Namikawa K Kobayashi I Ohba N Takahara Y Kadono C Tanaka A Kiyama H Targeted gene therapy toward astrocytoma using a Cre/loxP-based adenovirus system. Brain Res 2006 1081 34 43 16529724 10.1016/j.brainres.2006.01.105

[16] Nicholas TW Read SB Burrows FJ Kruse CA Suicide gene therapy with Herpes simplex virus thymidine kinase and ganciclovir is enhanced with connexins to improve gap junctions and bystander effects. Histol Histopathol 2003 18 495 507 12647801 10.14670/HH-18.495

[17] Wen Y Sachs G Athmann C A novel lens epithelium gene, LEP503, is highly conserved in different vertebrate species and is developmentally regulated in postnatal rat lens. Exp Eye Res 2000 70 159 68 10655141 10.1006/exer.1999.0770

[18] Wen Y Ibaraki N Reddy VN Sachs G Functional analysis of the promoter and chromosomal localization for human LEP503, a novel lens epithelium gene. Gene 2001 269 61 71 11376938 10.1016/s0378-1119(01)00439-5

[19] Malecaze F Lubsen NH Serre B Decha A Duboue M Penary M Berg D Arnaud JD Titeux M Kremer EJ Couderc B Lens cell targeting for gene therapy of prevention of posterior capsule opacification. Gene Ther 2006 13 1422 9 16724094 10.1038/sj.gt.3302790

[20] Yang J Liu TJ Lu Y Effects of bicistronic lentiviral vector-mediated herpes simplex virus thymidine kinase/ganciclovir system on human lens epithelial cells. Curr Eye Res 2007 32 33 42 17364733 10.1080/02713680601112793

[21] Yang J Lu Y Guo LH Liu TJ Mo XF The effect of lentivirus-mediated suicide gene therapy on lens epithelial cells. Zhonghua Yan Ke Za Zhi 2007 43 810 6 18070527

[22] Goto H Osaki T Kijima T Nishino K Kumagai T Funakoshi T Kimura H Takeda Y Yoneda T Tachibana I Hayashi S Gene therapy utilizing the Cre/loxP system selectively suppresses tumor growth of disseminated carcinoembryonic antigen-producing cancer cells. Int J Cancer 2001 94 414 9 11745423 10.1002/ijc.1474

[23] Greco O Joiner MC Doleh A Powell AD Hillman GG Scott SD Hypoxia- and radiation-activated Cre/loxP 'molecular switch' vectors for gene therapy of cancer. Gene Ther 2006 13 206 15 16307003 10.1038/sj.gt.3302640

[24] Kijima T Osaki T Nishino K Kumagai T Funakoshi T Goto H Tachibana I Tanio Y Kishimoto T Application of the Cre recombinase/loxP system further enhances antitumor effects in cell type-specific gene therapy against carcinoembryonic antigen-producing cancer. Cancer Res 1999 59 4906 11 10519403

[25] Nagayama Y Nishihara E Iitaka M Namba H Yamashita S Niwa M Enhanced efficacy of transcriptionally targeted suicide gene/prodrug therapy for thyroid carcinoma with the Cre-loxP system. Cancer Res 1999 59 3049 52 10397242

[26] Kanegae Y Lee G Sato Y Tanaka M Nakai M Sakaki T Sugano S Saito I Efficient gene activation in mammalian cells by using recombinant adenovirus expressing site-specific Cre recombinase. Nucleic Acids Res 1995 23 3816 21 7479022 10.1093/nar/23.19.3816 PMC307296

[27] Ueda K Iwahashi M Nakamori M Nakamura M Yamaue H Tanimura H Enhanced selective gene expression by adenovirus vector using Cre/loxP regulation system for human carcinoembryonic antigen-producing carcinoma. Oncology 2000 59 255 65 11053994 10.1159/000012169

[28] Krag S Andreassen TT Mechanical properties of the human posterior lens capsule. Invest Ophthalmol Vis Sci 2003 44 691 6 12556400 10.1167/iovs.02-0096

[29] Morgenbesser SD Williams BO Jacks T DePinho RA p53-dependent apoptosis produced by Rb-deficiency in the developing mouse lens. Nature 1994 371 72 4 8072529 10.1038/371072a0

